# Layer-by-layer decoration of MOFs on electrospun nanofibers

**DOI:** 10.1039/c8ra01260a

**Published:** 2018-03-15

**Authors:** Jinhong Shangguan, Lu Bai, Yang Li, Tao Zhang, Zhicheng Liu, Guizhe Zhao, Yaqing Liu

**Affiliations:** Shanxi Province Key Laboratory of Functional Nanocomposites, School of Materials Science and Engineering, North University of China Taiyuan 030051 China zcliu11@gmail.com lyq@nuc.edu.cn; School of Chemical Engineering and Technology, North University of China Taiyuan 030051 China bailu0919@gmail.com; Department of Mechanical Engineering, National University of Singapore Singapore 117574 Singapore

## Abstract

The design and fabrication of novel organic–inorganic nanocomposite membranes using metal–organic frameworks as building blocks have attracted numerous scientists. Here, HKUST-1 particles were decorated on crosslinked polymer nanofibers through a layer-by-layer method. The immersion sequence, the crosslinking and the number of the deposition cycles have a significant impact on the formation of the HKUST-1 decorated nanofibrous membranes. Moreover, it has been shown that such a membrane could be applied as a catalyst for visual detection of hydrogen peroxide.

## Abstract 2

A layer-by-layer method was introduced to fabricate MOF-decorated electrospun nanofibers, which could be used for visual detection of hydrogen peroxide.

## Graphical abstract

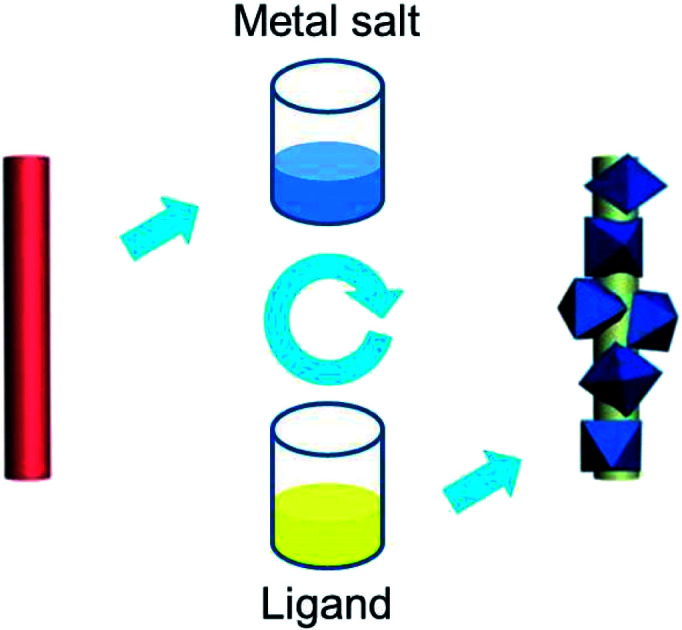

## 1. Introduction

Over the past two decades, metal–organic frameworks (MOFs), which are built from metal-based nodes and organic linkers, have been recognized as an emerging new type of porous material, which holds great potential for applications in adsorption, separation, catalysis, energy storage and conversion, sensing and drug delivery.^1–4^ The fascinating features such as highly tailorable nanostructures of the MOF materials have attracted considerable interest. However, because of their fragility, it is urgent to develop MOF composite materials to meet the requirements of practical devices.

An electrospun nanofiber membrane has acted as outstanding flexible three-dimensional template for the assembly of various nanomaterials since it provides a high surface area, highly porous nanostructure and large surface area to volume ratio.^5–7^ Since the pioneering work done by Hatton *et al.*, recent emerging MOF-assembled electrospun nanofibers have been designed and applied to many fields such as explosive detection, gas separation and light-emitting devices.^8–11^ Generally, there are two different strategies to fabricate such composite material. The first strategy is the assembly of *ex situ* synthesized MOFs in the nanofibers by mixing these MOFs with polymers in the precursor solution.^9–40^ For example, four kinds of MOFs were synthesized and mixed with three polymers such as polyacrylonitrile (PAN), polystyrene (PS), and poly(vinylpyrrolidone) (PVP) in solution by Wang *et al.*^31^ Then the mixture was electrospun into nanofibers, which showed impressive capability of air pollution control. Moreover, another group reported that different amounts of ZIF-8 MOF nanoparticles were incorporated into the poly(lactic acid) (PLA) to prepare the nanocomposite electrospun membrane.^25^ The obtained PLA/ZIF-8 electrospun nanofiber membranes exhibited increased oil wettability and improved mechanical property than the pure PLA membrane. It should be noted that the composite membrane with imbedded MOFs is not good at accessing external chemicals because of the polymer barrier, though these MOFs are well protected by the surrounding polymers. The second strategy is the *in situ* growth of MOFs on the electrospun nanofibers.^8,41–53^ Peterson and Parsons found that Zr-based MOF thin films could be formed *in situ* on the TiO_2_ coatings, which was deposited *via* atomic layer deposition onto polyamide-6 electrospun nanofibers.^51^ Another work presented by Ma and coworkers showed that bimetal ZIFs could directly grow on 2-methylimidazole/PAN electrospun nanofiber mats, which showed excellent electrocatalytic performance for oxygen reduction reaction after the carbonization step.^52^ Owing to the drawbacks such as time-consuming procedures and high cost of the existing methods, a facile and controllable method is urgently required for the design and fabrication of MOF nanocomposite membrane.

Here, HKUST-1, which is one of the most widely studied MOF materials, was used as model MOF to decorate poly(acrylic acid)/poly(vinyl alcohol) (PAA/PVA) electrospun nanofibers using a layer-by-layer method. Before the decoration, a simple and fast crosslinking process was applied to generate water-stable nanofibers with abundant active sites. The morphological evolution of the HKUST-1 decorated electrospun nanofibers was systematically investigated. Furthermore, the MOF nanocomposite membrane was utilized for the first time as a colorimetric platform for visual detection of hydrogen peroxide.

## 2. Experimental section

### 2.1 Preparation of PAA/PVA electrospun nanofiber membrane

PAA (*M*_w_ = 240 000, J&K Scientific) and PVA (PVA-2088, Shanghai Chenqi Chemical Science Co., Ltd) with weight ratio of 4 : 1 were dissolved in water by stirring for 12 h to form a 25 wt% transparent solution. In a typical electrospinning process, the PAA/PVA aqueous solution was pumped through a syringe pump at a flow rate of 0.3 mL h^−1^. The applied voltage was kept at 15 kV, while the distance between the needle tip and the collector was 15 cm. The PAA/PVA electrospun nanofiber membrane was collected and then dried in a vacuum oven at room temperature. The obtained nanofibers were crosslinked upon a simple heating treatment at 145 °C for 15 min.

### 2.2 Layer-by-layer decoration of MOFs on electrospun nanofibers

The growth of the HKUST-1 MOFs on the PAA/PVA electrospun nanofibers was accomplished using a layer-by-layer method. Briefly, each growth cycle consisted of the immersion of the nanofibers in the 0.05 M copper(ii) acetate (Cu(OAc)_2_, Sinopharm Chemical Reagent Co., Ltd) aqueous solution for 2 h and the immersion of the nanofibers in the 0.05 M 1,3,5-benzenetricarboxylic acid (BTC, Sinopharm Chemical Reagent Co., Ltd) ethanol solution for 2 h. The membrane was rinsed with water and ethanol after each immersion step. In order to control the whole growth process of dense MOFs-decorated nanofiber membrane, different growth cycles were performed.

The HKUST-1 powder sample was prepared by direct mixing 10 mL of 0.15 M Cu(OAc)_2_ solution with 10 mL of 0.1 M BTC solution under stirring. The reaction was allowed to proceed for 24 h, and then the HKUST-1 particles were collected by centrifugation at 10 000 rpm for 10 min. Finally, the particles were washed with ethanol and dried in an oven.

### 2.3 Visual detection of hydrogen peroxide

1 mg of HKUST-1 decorated nanofiber membrane was added into 4 mL PBS buffer solution (pH = 7), followed by adding 100 μL of 50 mM *o*-phenylenediamine (OPD, Sinopharm Chemical Reagent Co., Ltd) solution and 150 μL of 50 mM hydrogen peroxide (H_2_O_2_, Sinopharm Chemical Reagent Co., Ltd). The mixed solution was incubated at 75 °C for 30 min. Then the optical spectrum of the solution was recorded using an UV-vis spectrophotometer (Unico UV-4802).

### 2.4 Characterization

The morphologies of the nanofiber membranes were observed using scanning electron microscopy (SEM, Hitachi SU8010). The X-ray diffraction (XRD) patterns were acquired from a DX-2700 X-ray diffractometer. The thermogravimetric (TG) measurements were performed on a TA Q 50 thermogravimetric analyzer. Fourier-transform infrared spectroscopy (FTIR) spectra were collected with a Nicolet IS50 spectrometer using an attenuated total reflection module. The specific surface area of the nanofiber membranes were measured by a Micromeritics ASAP-2460 accelerated surface area and porosimetry system.

## 3. Results and discussion

**Fig. 1:**
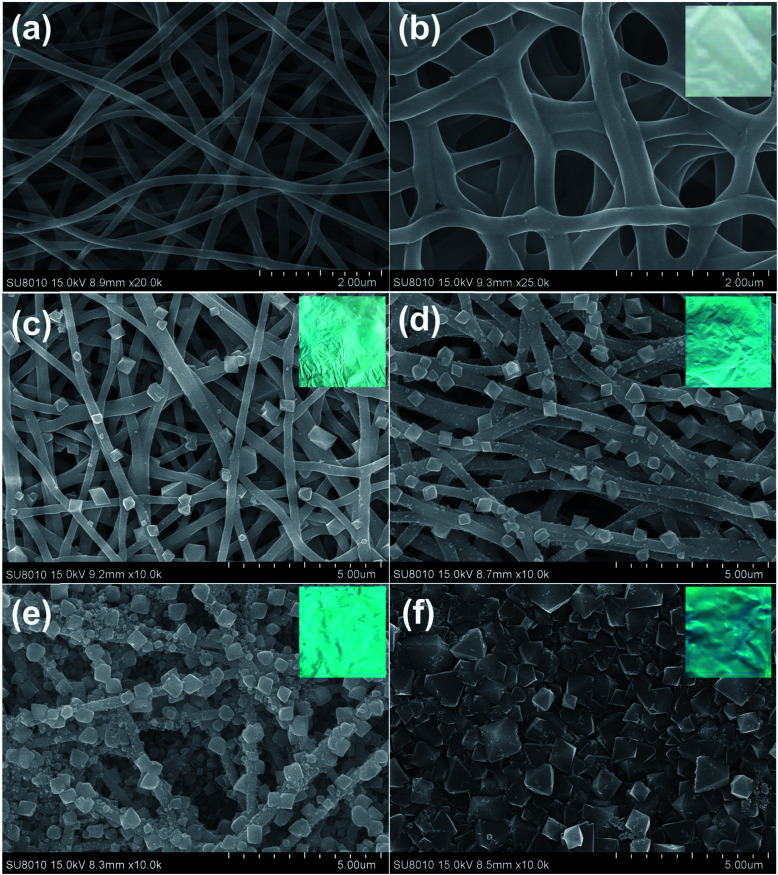
SEM images of the pristine PAA/PVA nanofibers (a), the crosslinked PAA/PVA nanofibers (b), MOF-decorated nanofibers after 1 cycle of growth (c), MOF-decorated nanofibers after 2 cycle of growth (d), MOF-decorated nanofibers after 4 cycle of growth (e) and MOF-decorated nanofibers after 8 cycle of growth (f). The insets are the corresponding photos of the membranes.

It is worth to mention that the *in situ* growth strategy requires both stable nanofibers as the template and numerous active groups (*e.g.*, hydroxyl and carboxyl) to anchor the MOFs. PAA/PVA nanofibers were selected as template for the growth of HKUST-1 because they can provide lots of binding sites for the metal ions. However, the nanofiber membrane is soluble in aqueous solution owing to the usage of water-soluble polymers. A facile thermal treatment was introduced to crosslink the carboxylic acid of PAA and the hydroxyl groups of PVA, resulting in water-stable PAA/PVA nanofibers.^54,55^ As shown in Fig. 1a and b, the diameter of the crosslinked nanofibers (about 250 nm) was a bit larger than that of the pristine nanofibers (about 150 nm), while the three-dimensional porous feature of the membrane was retained. This nanofibrous nanostructure benefits the access of the metal ions and the ligands in the growth solution.

The deposition of HKUST-1 on the nanofibers was accomplished by repeated dipping the nanofiber membrane first in Cu(OAc)_2_ solution and then in BTC solution. After one cycle of growth, a few typical octahedral HKUST-1 crystals were observed on the surface of the nanofibers (Fig. 1c). More and more MOF particles were decorated on the nanofibers by increasing the deposition cycles (Fig. 1d and e), and finally a dense film fulfilled with HKUST-1 particles was achieved after eight cycles of growth (Fig. 1f). The size of the HKUST-1 crystal also increased with the increasing deposition cycles. Moreover, the color of the membrane changed from white to blue, implying the successful decoration of HKUST-1 (the insets of Fig. 1). It is worth mentioning that the layer-by-layer growth allows the formation of uniform MOF-decorated electrospun nanofibers over a large area (*e.g.*Fig. 1e), which might facilitate the fabrication of large area devices.

**Fig. 2:**
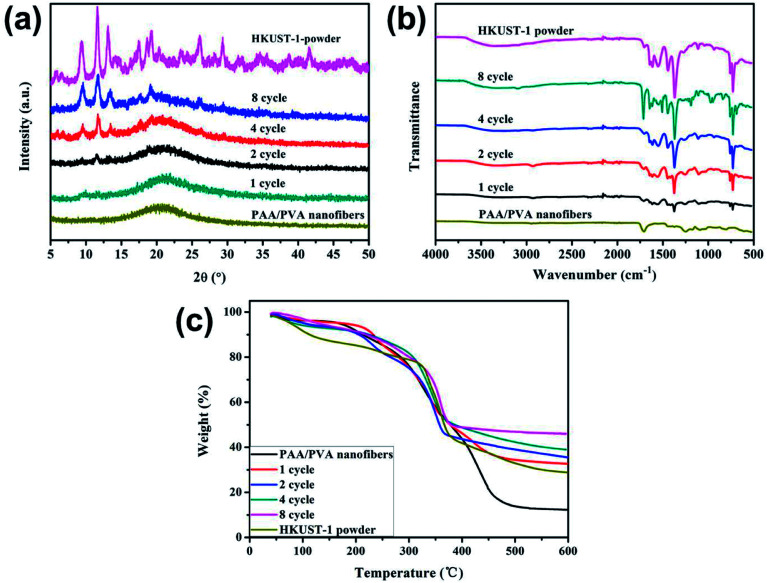
XRD patterns (a), FTIR spectra (b) and TG curves (c) of the PAA/PVA nanofibers, the HKUST-1 powder and the HKUST-1 decorated nanofibers after different cycles of growth.

The decoration of HKUST-1 on the nanofibers was further analyzed using multiple techniques. The XRD pattern of the PAA/PVA nanofibers shows an amorphous structure, while the patterns of the MOF decorated nanofibers contain characteristic diffraction peaks of HKUST-1 (Fig. 2a), suggesting the formation of well-crystallized MOF structure.^14^ Further confirmation of the formation of the nanocomposite membrane is provided by the FTIR spectra presented in Fig. 2b. Both the typical vibration bands of HKUST-1 located at 729, 759, 1370 and 1714 (carbonyl stretching, also showed in PAA/PVA nanofibers) cm^−1^ and the vibration of –CH_2_– group of the polymer nanofiber located at 1246 cm^−1^ were clearly observed for all MOF-decorated nanofiber membranes.^46^ What is more, the TGA tests suggest that the content of the residue increases with the increasing content of the decorated HKUST-1 (Fig. 2c). Since porous MOF materials were assembled on the electrospun nanofibers, it is expected that the surface area would intensively increase. The Brunauer–Emmett–Teller (BET) surface area of the HKUST-1 decorated nanofibers increased from 25.7 m^2^ g^−1^ after one cycle of growth to 227.7 m^2^ g^−1^ after eight cycles of growth, while the BET surface area of the PAA/PVA nanofibers is 1.9 m^2^ g^−1^. It is believed that such MOF membrane could be applied to gas adsorption and separation.

**Fig. 3:**
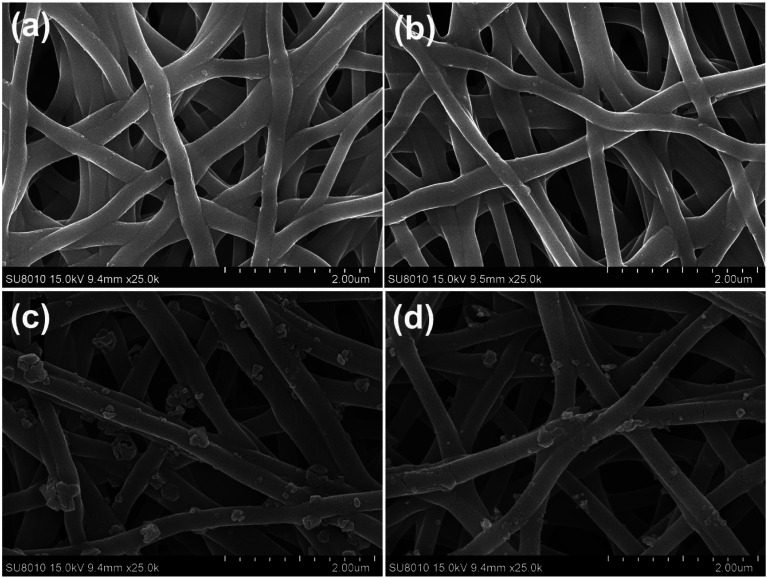
SEM images of various nanofiber membranes. (a) The nanofibers were immersed only into the metal ion solution for 2 h; (b) the nanofibers were immersed into the ligand solution first and then into the metal ion solution; the decoration of HKUST-1 on the nanofibers after crosslinking for 30 min (c) and 1 h (d), and the growth process was identical with the sample in Fig. 1c (after crosslinking for 15 min).

In order to get more insight into the growth process, complementary experiments were performed. Fig. 3a shows that none HKUST-1 particles would form without the BTC ligand. When the nanofibers were immersed into the ligand solution first and then into the metal ion solution (Fig. 3b), there was no decoration of MOFs on the nanofibers, indicating that the active sites on the PAA/PVA nanofibers should bind the metal ion first through electrostatic interaction or chelation. Moreover, the crosslinking process could efficiently affect the amount of the active groups since the active groups of the polymers take part in the crosslinking, resulting in less decoration of HKUST-1 on the nanofibers with increasing crosslinking time (Fig. 3c and d).

**Fig. 4:**
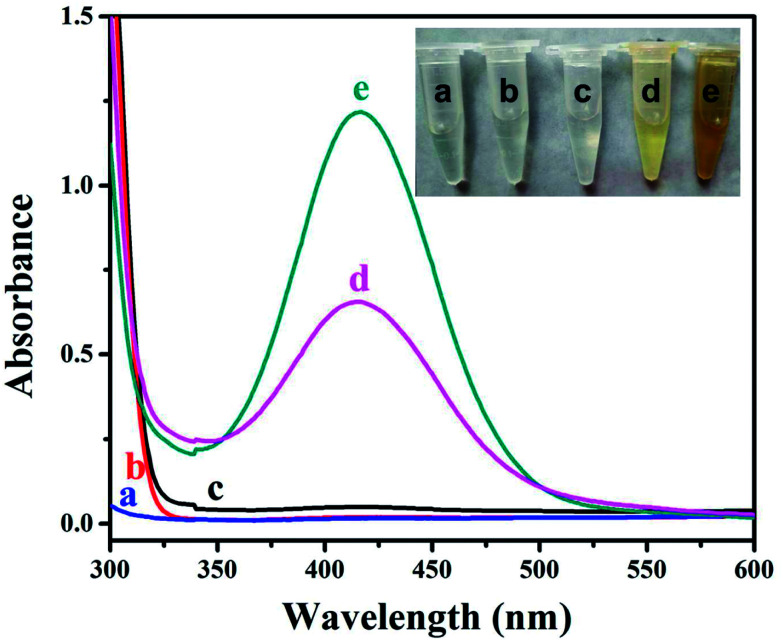
UV-vis spectra of the solutions consisted of (a) OPD and H_2_O_2_, (b) HKUST-1 decorated nanofiber membrane and H_2_O_2_, (c) OPD, (d) OPD and HKUST-1 decorated nanofiber membrane, (e) OPD, H_2_O_2_ and HKUST-1 decorated nanofiber membrane. The inset is the corresponding photo of the solutions.

**Fig. 5:**
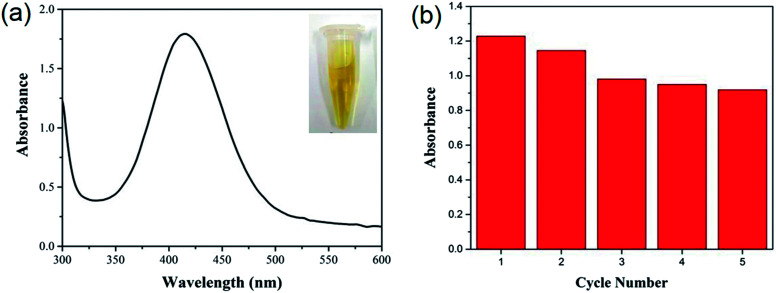
(a) UV-vis spectrum of the mixed solution of OPD and H_2_O_2_ after the addition of 100 μL of 1 mM Cu(OAc)_2_ solution; (b) the peak intensity at 418 nm was recorded to test the recyclability of the HKUST-1 decorated nanofiber membrane for the detection of H_2_O_2_.

Owing to its extreme simplicity and low cost, visual detection of target analyte, which is based on color changes observed by naked eyes, has received a lot of attention. It is recently reported that some MOFs show peroxidase-like catalytic activities, catalyzing the oxidation of *o*-phenylenediamine (OPD) with obvious color change of solution in the presence of hydrogen peroxide (H_2_O_2_).^56,57^ As a proof of concept, the visual detection of H_2_O_2_ using HKUST-1 decorated nanofiber membrane as catalyst is demonstrated here. As shown in Fig. 4, no absorption was noticed for the solutions consisted of OPD and H_2_O_2_, while the solution consisted of OPD, H_2_O_2_ and HKUST-1 decorated nanofiber membrane turned brownish yellow, which was eye-catching. The oxidation of OPD might be mediated by the Cu^2+^ ions in HKUST-1 because the PAA/PVA nanofiber membrane did not show catalytic activity (Fig. 5a). The Cu^2+^ ions might act as a peroxidase mimic to the oxidation of OPD, and they might originate from the dissolution of the HKUST-1 particles. It is notable that the dissolved oxygen may also act as oxidant, resulting in light yellow solution after the addition of HKUST-1 decorated nanofiber membrane.^58^ Nevertheless, a novel platform based on the peroxidase-like activity of the HKUST-1 decorated nanofiber membrane was successfully established for visual detection of H_2_O_2_. Moreover, this MOF-decorated nanofiber membrane could be readily recycled and reused for at least five times (Fig. 5b), which is superior to other MOF powders.^56,57^

## 4. Conclusions

HKUST-1 particles could be decorated on the surface of the crosslinked PAA/PVA nanofibers *via* alternative immersion into metal ion solution and ligand solution. This controllable layer-by-layer method is easy-to-process and cost-efficient. The surface area of the HKUST-1 decorated nanofiber membrane was 227.7 m^2^ g^−1^ after eight cycles of growth. Furthermore, the HKUST-1 decorated nanofiber membrane could be used for the visual detection of H_2_O_2_. It is believed that such designed MOF-decorated nanofiber membranes may have a promising future in many fields such as gas adsorption, hazardous removal and catalysis.

## Conflicts of interest

There are no conflicts to declare.

## Supplementary Material
